# Qualitative study of paramedics' experiences of managing seizures: a national perspective from England

**DOI:** 10.1136/bmjopen-2016-014022

**Published:** 2016-11-09

**Authors:** Adam J Noble, Darlene Snape, Steve Goodacre, Mike Jackson, Frances C Sherratt, Mike Pearson, Anthony Marson

**Affiliations:** 1 Department of Psychological Sciences, University of Liverpool, Liverpool, UK; 2 School of Health and Related Research, University of Sheffield, Sheffield, UK; 3 North West Ambulance Service NHS Trust, Bolton, UK; 4 Department of Molecular and Clinical Pharmacology, University of Liverpool, Clinical Sciences Centre, Liverpool, UK; 5 Aintree Health Outcomes Partnership, University of Liverpool, Clinical Sciences Centre, Liverpool, UK

**Keywords:** ACCIDENT & EMERGENCY MEDICINE, EDUCATION & TRAINING (see Medical Education & Training), QUALITATIVE RESEARCH

## Abstract

**Objectives:**

The UK ambulance service is expected to now manage more patients in the community and avoid unnecessary transportations to hospital emergency departments (ED). Most people it attends who have experienced seizures have established epilepsy, have experienced uncomplicated seizures and so do not require the full facilities of an ED. Despite this, most are transported there. To understand why, we explored paramedics’ experiences of managing seizures.

**Design and setting:**

Semistructured interviews were conducted with a purposive sample of paramedics from the English ambulance service. Interviews were transcribed and thematically analysed.

**Participants:**

A diverse sample of 19 professionals was recruited from 5 different ambulance NHS trusts and the College of Paramedics.

**Results:**

Participants’ confirmed how most seizure patients attended to do not clinically require an ED. They explained, however, that a number of factors influence their care decisions and create a momentum for these patients to still be taken. Of particular importance was the lack of access paramedics have to background medical information on patients. This, and the limited seizure training paramedics receive, meant paramedics often cannot interpret with confidence the normality of a seizure presentation and so transport patients out of precaution. The restricted time paramedics are expected to spend ‘on scene’ due to the way the ambulance services’ performance is measured and that are few alternative care pathways which can be used for seizure patients also made conveyance likely.

**Conclusions:**

Paramedics are working within a system that does not currently facilitate non-conveyance of seizure patients. Organisational, structural, professional and educational factors impact care decisions and means transportation to ED remains the default option. Improving paramedics access to medical histories, their seizure management training and developing performance measures for the service that incentivise care that is cost-effective for all of the health service might reduce unnecessary conveyances to ED.

**Strengths and limitations of this study**

- This is the first study to explore from a national perspective paramedics’ views and experiences of managing seizures.
- Paramedics from five different ambulance services were recruited and so it is likely the issues reported do not relate to isolated, local concerns, but reflect practice across the country.
- Our results may have relevance internationally as countries such as the USA, Australia, Canada and New Zealand have similarly organised emergency care systems and are also seeking new ways to reduce conveyance rates and emergency department admissions.
- Our study is based on the perceptions and experiences of a self-selecting sample of participants, rather than observations of what the actual barriers are.
- The study also did not capture the perspective of associated services providers (eg, urgent care centres, general practitioners), nor patients and carers.

## Introduction

Ambulances frequently attend to people who have experienced a suspected seizure.^1^ Seizures can be provoked by a number of causes; some are life-threatening. However, in most instances, the patient will be someone with a known epilepsy diagnosis, experiencing an uncomplicated seizure. While some postictal drowsiness and confusion is common, the full facilities of a hospital emergency department (ED) are not required.^2–4^ It is therefore concerning that recent UK-wide National Audits of Seizure Management in Hospitals found most visits to ED for seizures *are* by those with known rather than new epilepsy and for uncomplicated seizures.^5^ Similar patterns of use are seen in other countries.^6^ ^7^

Reducing unnecessary visits to EDs for seizures has been identified as one way that resource-limited health services can generate savings.^8^ In England alone, there are around 100 000 visits to EDs for epilepsy each year.^5^ The cost of providing this care in 2012/2013 was >£56 million.^9^ The ambulance service has a critical role in helping achieve any reduction, as nearly all seizure patients (∼90%) attending ED arrive by emergency ambulance.^10^ While the UK ambulance service—like those in the USA, Canada and Australia—has traditionally been viewed as a ‘call-handling and transportation service’,^11^ this is no longer the case. Paramedics are not obliged to convey all patients they see to ED; rather, they are expected, where appropriate, to treat more patients ‘at scene’ and refer to alternative, non-emergency care pathways.^12–14^

Despite this, paramedics still transport most seizure patients to ED.^1^ ^15^ One regional English ambulance service reported that in only 19% of seizure cases is the patient *not* conveyed.^15^ Understanding why this is the case is difficult as almost no information is available on how paramedics experience managing seizure patients and make decisions about the care they offer.

Only one study to date has considered the issue;^16^ for it, one of us (AJN) recruited and interviewed 15 ambulance clinicians. Results indicated that patients with epilepsy can be taken to ED after a seizure not because of clinical need, but because the attending clinician does not feel sufficiently confident or informed to be able to adequately assess patients’ medical needs. Only around half said they were confident managing seizures. This was compounded by a perceived lack of alternatives to ED conveyance for necessary continued care, as well as fears over litigation if they did not convey a patient and an adverse event occurred.

The previous study was limited in that participants were recruited from a single, urban service and so the results may not be generalisable. It is also not clear what impact on practice the recent sharp increase in demand for the ambulance service has had. Over the last 5 years, calls to the service have increased by 15%.^17^ ^18^

**Figure 1:**
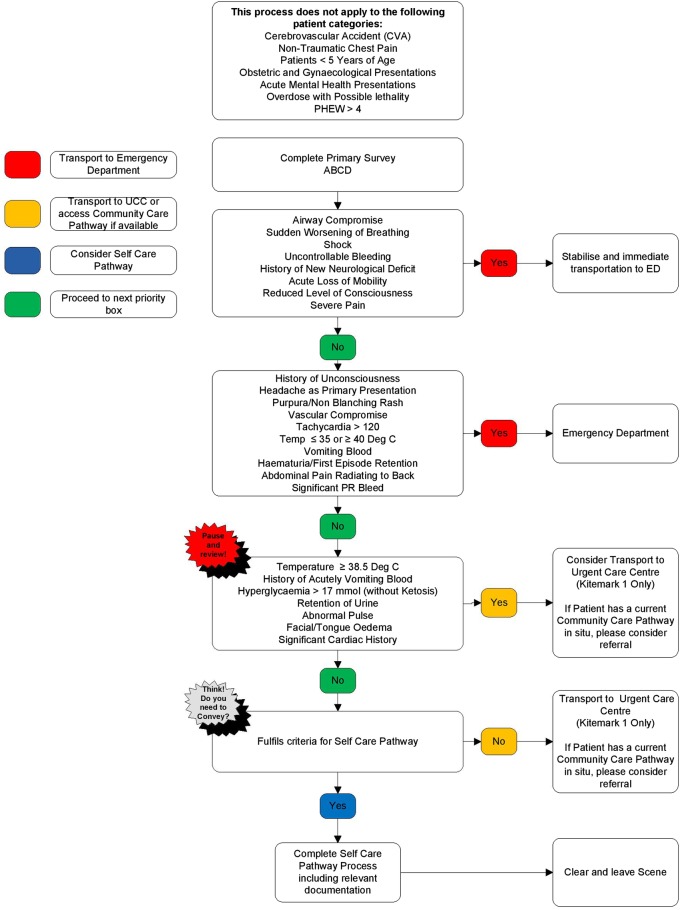
Paramedic Pathfinder tool for medical patients (reproduced with permission of North West Ambulance Service). ABCD, airway, breathing, circulation, disability; PHEW, prehospital warning score; PR, perirectal; UCC, urgent care centre; ED, emergency department.

Second, the study did not clarify paramedics’ use of the guidelines and tools made available to them. Ambulance services in the UK are guided by the Joint Royal Colleges Ambulance Liaison Committee's (JRCALC) national guidelines (table 1).^19^ Some organisations have also recently made available to staff versions of a generic triage support tool called ‘Paramedic Pathfinder’ (figure 1).^20^ It has been contended^20^ that this tool should facilitate non-conveyance as, based on a patient's symptoms and vital signs, it categorises patients by the nature of onward care they require.

**Table 1:** Overview of 2016 JRCALC^19^ national guidance regarding who should and should not be transported to emergency department

	Guidance
Transfer to further care	Patients suffering from serious convulsions (≥3 in an hour) Patients suffering from eclamptic convulsions Patients suffering their first convulsion Difficulties monitoring the patient's condition
Non-conveyance	Only consider leaving a patient at home who makes a fully recovery following a convulsion if they are known to suffer from epilepsy, and can be supervised adequatelyFor these patients: Measure and record vital signed with explanation given to the patient Advise patients/carer to contact GP if patient feels generally unwell or call ‘999’ if there are repeated convulsions Document reasons for decision and this must be signed by patient and/or carer Provide an information leaflet Ensure contact is made with the patient's GP Consider referral to local epilepsy service for review/ follow-up.
	

GP, general practitioner; JRCALC, Joint Royal Colleges Ambulance Liaison Committee.

Finally, our prior study appeared to raise the possibility that additional training in seizure management may be of value to many of the 20 000 paramedics operating in the UK.^21^ It did not, however, explore the views of paramedics about this, its required content, uptake or likely effect.

Given this, the current project explored the experiences of paramedics from across England when it comes to managing seizures. This information could help better understand how the ambulance service might reduce unnecessary and costly conveyances to ED. We aimed to identify what, if any, challenges paramedics experience when managing seizure presentations, what their support needs were, including educational, and what their views were of the utility of tools such as the JRCLAC guidelines and Pathfinder. To do this, we recruited and completed detailed interviews with paramedics from across the country. We here present evidence on the experiences of paramedics of managing seizures and the factors influencing their care and conveyance decisions. In a second article, we present findings on paramedics’ views of seizure management training for practicing clinicians (FC Sherratt, D Snape, S Goodacre, *et al*. Paramedics’ views on their seizure management learning needs: a qualitative study in England. BMJ Open submitted).

## Methods

### Design

Semistructured qualitative interviews were conducted with representatives from the ambulance service. This data collection approach was best suited to our aims as it provides a medium by which the world can be understood from a participant's point of view.^22^ Participants are able to raise what they regard as important issues and concerns, rather than the researcher imposing predetermined structures and assumptions.^23^

The interviews were introduced to participants as looking to explore paramedic's views regarding seizure management and what, if any, were their support needs. Following Riessman,^24^ an interview topic guide was developed on the basis of the literature and refined through the iterative process of conducting two pilot interviews (table 2).^25^ Areas covered included: (1) an introductory phase; (2) participants perceptions of the challenges crews face when managing seizures; (3) availability of discharge options for persons who did not need to be conveyed to ED; (4) training and/or support offered to crews from their organisation; (5) potential strengths and weakness in relation to the assessment tools provided to paramedics by the service and (6) views in relation to additional training needs on seizure management for paramedics. The use of the topic guide, primarily as a conversational agenda rather than a procedural directive,^26^ enabled the researcher to clarify uncertainties with follow-up questions and to use the responses given by participants to continually inform the evolving conversation.^23^ ^27^

**Table 2:** Overview of sections of interview topic guide relevant to this current report and interviewer

Following a brief introduction and the participant being asked about their background and role, they were asked about their views of and experiences of managing seizures. The main themes relevant to this current report with examples of prompts are given below:	
Theme	Example questions
Perceptions of challenges faced when managing seizure	What are the main challenges you perceive ambulance crews face in managing seizures? What factors influence care-decisions? Why? What sort of confidence do you/paramedics have in managing seizures? What accounts for this?
Discharge options for persons who did not need ED	What options are available when a person does not need to be conveyed to ED? What are your experiences and views of using and accessing these?
Potential strengths/weakness in relation to support offered to crews	In what way are paramedics supported in their clinical decision-making by their organisation (eg, on-scene/after-scene support/protocols/guidance)? What are your experiences/views of these?

ED, emergency department.

FCS (PhD), a university-based qualitative researcher with a specialist interest in health services research but no specialist knowledge of the ambulance service, conducted the interviews. Participants were informed that participation was anonymous and told the study results would be published. No non-participants were present during the interviews. To promote transparency, meticulous records of the interviews were kept, interviews audio-taped and subsequently transcribed verbatim.^28^ To help validate the data, participants were also offered the opportunity to comment on their interview transcript (member checking).^25^

### Recruitment and setting

The English ambulance service comprises 10 regional NHS Ambulance Trusts, with separate arrangements for the Isle of Wight (table 3). Most (65%) practicing paramedics are aged between 30 and 49 years and male (62.0%), with the gender difference being most pronounced within managerial positions (77.0%).^42^ Paramedics have traditionally trained through inservice training routes provided by ambulance services—the Institute of Health and Care Development paramedic programme (IHCD).^44^ A degree-level qualification has only become an option in recent years.

**Table 3:** Characteristics of the different regional ambulance services operating within England

Service	Population covered*	Square miles covered*	Number of qualified ambulance staff (FTE)†,‡	Calls to which an emergency response was dispatched§,¶	Proportion seen, but not conveyed to ED§^,^**	Recruitment site?
London Ambulance Service NHS Trust	8.6 million	620	2597	1 047 357	34.4	✓
North West Ambulance Service NHS Trust	7 million	14 000	2852	878 352	30.9	✓
East of England Ambulance Service NHS Trust	6 million	7500	1688	697 901	41.6	–
West Midlands Ambulance Service NHS Foundation Trust	5.6 million	5000	2201	838 069	37.3	–
Yorkshire Ambulance Service NHS Trust	5 million	6000	1540	521 331	31.1	✓
East Midlands Ambulance Service NHS Trust	4.8 million	6425	1484	542 325	33.6	✓
South East Coast Ambulance Service NHS Foundation Trust	4.6 million	3600	1592	656 338	45.3	–
South Central Ambulance Service NHS Foundation Trust	4 million	3554	1041	445 798	42.0	–
North East Ambulance Service NHS Foundation Trust	2.7 million	3200	642	297 826	32.5	–
South Western Ambulance Service NHS Foundation Trust	2.5 million	5000	1875	599 189	52.4	✓
Isle of Wight NHS Trust	140 000	147	60	19 683	51.8	–

*Information from the following sources: refs. 29–41

**†**Information from ref. 42.

**‡**Qualified ambulance staff here includes paramedics, technicians, advanced practitioners and ambulance service managers but does not include ambulance trainees.

§Taken from ref. 43.

¶Face-to-face responses as a result of 111 calls.

**Treatment at the scene or onward referral to an alternative care pathway and those with a patient journey to a destination other than ED.

We aimed to recruit a sample of 20 paramedics. To increase reliability, we wanted the sample to be geographically diverse. To do this, we therefore sent advertisements to members of the ‘National Ambulances Leads’ group which has representation from each of the 10 ambulance services. They were each asked if their service would be a recruitment site for the study. Five services ultimately agreed and are highlighted in table 3. In 2015/2016, they were responsible for providing emergency care for 28 million residents in England (50% of the population) and collectively received 3.8 million emergency calls.^43^ They include the largest, busiest and most rural services, as well as the ones which have historically transported the highest and lowest proportions of their patients to ED (range 70–48%).^43^

Sampling was purposive, consisting of a group of informed individuals/‘experts’ deemed to have a high level of knowledge and clinical experience of paramedic policy and practice. To reduce possible bias, sampling reflected the specialisms under investigation. Ambulance sites were asked to circulate the advert among their respective educational, consultant and advanced paramedic teams or similar. This approach aimed to permit the recruitment of a diverse sample of professionals, so that a range of perspectives could be captured and inform analysis. We considered persons within the stated teams as being positioned to provide a sufficient overview of various aspects of the service. The College of Paramedics, the professional body for the service in the UK, was also invited to identify a representative from its educational team to be interviewed. We excluded emergency care technicians and assistants from the sample, as it is paramedics, as the registered health professional on board most ambulances, who typically lead care management decisions.

Informed consent was obtained from all participants.

### Data analysis

Thematic analysis, informed by the work of Braun and Clarke,^45^ was used to enable scrutiny of data across the sample and within individuals’ transcripts and professional roles. It was conducted deductively with the identification of pre-existing themes underpinned by previous research and inductively with the identification of themes grounded in the data^46^ to identify patterns and themes related to the study objectives.

FCS led the analysis process and was supported by AJN and DS. Familiarity with the data was developed through repeated listening of the audio-tape(s) and line-by-line reading of the transcripts. FCS read each transcript, AJN the first 10 and DS the remaining 9. Each independently made notes summarising significant events and themes of interest; a process similar to ‘memo-ing’ in grounded theory.^47^ During analysis, verification of emerging findings and interpretations was conducted via five research team meetings. These discussions offered fresh insight—personal, professional and methodological—and enabled FCS to reflect on potential biases and assumptions.

QSR International’s NVivo V.10^48^ qualitative data analysis software was used as a management tool throughout the process. The purpose of this was to provide a transparent account of our work and ensure a rigorous approach to data analysis. The powerful search facilities of this software enabled the identification of key words, phrases and attributes from across the data set and allowed examination of data from a number of different perspectives. Nodes were created to mark relevant concepts and topics in the text documents. Relationships between themes were identified through constant comparison of the transcripts, nodes and categories. An account of the process of analysis was logged in the memos attached to categories and interview documents.

Quotations are presented to illustrate themes. There has been minor editing of some to preserve anonymity and to ensure clarity of meaning.

## Results

### Participants

Nineteen participants were recruited and interviewed. This consisted of 18 paramedics from 5 regional ambulance services and a paramedic from the educational section of the professional body. The interviews took place between January and March 2016. Their average duration was 70 min (range 47–116). Sixteen interviews were conducted by telephone and three were face-to-face. One additional paramedic volunteered to participate within but was not ultimately recruited because they found themselves to be too busy.

The majority of participants were male (n=15), with the mean ambulance service experience of 20 years (SD=9.6). Most (n=17) had entered the profession via an ambulance service's IHCD programme rather than a Higher Education Institution route (table 4). Paramedics’ role titles differed between ambulance trusts. Because of this, and to protect participants’ anonymity, role titles were collapsed into role specialisms. Specialisms were guided by the Paramedic Career Framework developed by the College of Paramedics.^49^

**Table 4:** Participants’ characteristics

Participant	Gender	Approximate ambulance service experience (years)	Paramedic training route	Role specialism
1	Female	10	HEI	Clinical
2	Male	18	AT	Clinical
3	Male	22	AT	Clinical
4	Female	15	AT	Clinical
5	Male	25	AT	Clinical
6	Male	14	AT	Management
7	Male	6	AT	Education
8	Male	32	AT	Management
9	Male	19	AT	Clinical
10	Male	33	AT	Education
11	Female	11	HEI	Management
12	Male	21	AT	Clinical
13	Female	8	AT	Management
14	Male	21	AT	Education
15	Male	22	AT	Clinical
16	Female	24	AT	Education
17	Male	18	AT	Clinical
18	Male	45	AT	Education
19	Male	12	AT	Education

AT, ambulance trust; HEI, higher education institute.

### Themes

Analysis of the transcripts provided insights into the main seizure presentation paramedics encountered. It also identified a range of challenges faced when managing such patients and how these impacted on conveyance decisions. These were for the most part common across the services recruited from and other categories, including sex. Four key themes were identified: (1) need for relevant historical information to guide care and conveyance decisions; (2) perverse incentives to convey to ED caused by time pressure/performance requirements; (3) knowledge gaps and uncertainty about postictal care and (4) limitations in care pathways and need for patient-centred care. These shall now be expanded on in turn, with further illustrative quotes being are provided in table 5.

**Table 5:** Themes within participant interviews and quotes illustrating them

Theme	Subtheme	Illustrative quotes
Need for relevant historical information to guide care and conveyance decisions	Information gaps about patients prior history	The biggest challenge begins with seizures themselves because more often than not when a crew arrives the seizure has ceased so you're relying entirely on individuals present to describe things to you… If you've only got the individual that suffered the seizure present while they're in the recovery phase it's very difficult to establish a full history. You've got to think about whats caused it … it might be epilepsy, but you've got to think about hypoxia, hyperglycaemia, is there any sort of toxic issues going on. (p. 4) While they're postictal we don't know if it's a normal fit for them, we don't know when they last had one, we don't know if they've had a history of repeated ones. We don't even know if they are epileptic or not half the time. (p. 3)
	Obtaining information is challenging	Finding a patient with medical alert band on them to say that they've got epilepsy or carrying a seizure diary is like ‘striking gold’…most of the time you're guessing really what's normal for the patient. You are trying to pick it up and work it out as you go along and you reach a sort of limit of what you can access, especially if the patient remains postictal while they're in your care… (p. 1) You can try to ring a patient's GP for further information, but I'll tell you now that doesn't help…Just to get to speak to a GP is nigh on impossible. They're extremely busy. (p. 8) You can have to wait for GPs or out of hours doctors to phone you back…we can wait an hour and half. (p. 13)
Perverse incentives to convey to ED caused by time pressure/performance requirements	Times pressure can impact care decisions	There's an expectation that we will turn a job around you know…if they've been on scene for 20/30 min crews start to feel almost panicky that they're taking up time and that they need to get on with it. (p. 4) If someone has a seizure outside of the home, we wouldn't really take them home… It's not necessarily the right option for that patient… But by taking them home, which is further away, we will be tied up for longer. (p. 6) If I've been on scene a while I'll get messages sent down the mobile data terminal saying ‘Are you ok?’ which is a euphemism for ‘why is it taking you so long?’ So again it's that idea of we'll just put them in the back of the ambulance while their postictal and start driving to hospital rather than waiting to see if they recover… (p. 3)
	Time pressures operate differently in rural areas	Large urban areas are saturated with hospitals and if I'm getting monitored and measured on time performance well I might as well just take all my epileptic patients to hospital because I'm only 4 min away…I've done my job, the patient's safe and I've hit my time targets, I'm not going to be criticised by anybody. Whereas where we work in a more rural setting…that's not the case. (p. 8)
Knowledge gaps and uncertainty about postictal care	Limited training on seizures for paramedics	Epilepsy and convulsions don't come into any post-registration training… I have not had a single days training in managing convulsions since I was first trained in 1987. (p. 8) Paramedic training is geared towards critical illness, critical injury but we're seeing less and less of that and we're seeing more and more of chronic illness. (p. 12)
	Knowledge and confidence in seizure management low	There certainly needs to be more training on epilepsy because hand on heart I think if you took most ambulance crews today and said tell me about epilepsy, tell me what's going on, tell me about serial convulsions, tell me about status epilepticus, tell me about eclampsia and how would you recognise that from somebody having an epileptic convulsion, I think you would start hitting boundaries, I really do. (p. 8)
Limitations in care pathways and need for patient centred care	Pathfinder offers some reassurance and structured decision-making	If we follow that (Paramedic Pathfinder), the Trust will support us in our decision-making… so if something were to go wrong and we've used Pathfinder, that supports us. (p. 6)
	For most JRALC and pathfinder are unhelpful	There's] only one paragraph in JRCALC that relates to patients who've had a seizure…it's very vague, it's definitely left to your own clinical interpretation about what you feel is safe. (p. 1) We have got the Pathways flowcharts. But if you do too much of that it becomes ambulance service by numbers. Indeed some of the statements are quite vague, such as the one about whether the patient has a history of unconsciousness. You might say well ‘look a person's who's had a seizure will have had a history of unconsciousness and therefore now I'm going to transport because that's what the pathway dictates’… I've found this. (p. 7)
	Fear of adverse events if patient is not conveyed	They worry the patient is going to have another convulsion. How do they differentiate between the patients that need to go to hospital vs the patients that don't?…I think the service would support you [if an adverse event occurred] but I think over half of staff think they won't be… its of a lack of information about what actually happens the vast majority of times. (p. 3) ‘the paramedics will think in the back of their mind if I discharge this person on scene and allow them to continue their journey to work or wherever and they suffer another seizure and fall under a train, I will be responsible for that as the last practitioner to have seen that patient’…so some staff might well ask, ‘Non-conveyance of patients, what's really in it for me?’ (p. 9) A lot of clinicians and it's a historical thing have this perception that if they leave a patient and something then goes wrong erm that that the ‘book will be thrown at them’, that you know it will be it will be deemed to be their fault… (p. 4)
	Lack of alternative care pathways	We struggle for alternative pathways and so while we might be directed towards primary care, when we actually try and put some of those pathways into actual practice, they do seem to be lacking. (p. 7) So we have a self-care pathway option for epileptics. The patient must be over the age of 16, be an known epileptic and there are a number of criteria. One is that there must be a competent carer or individual who can accept responsibility of care for the patient…that's one that we quite often fail on especially within a public place. (p. 5) I think it just comes back to those challenges of I'm worried I might make mistake, I'm worried that they might get worse, I'm I haven't got anyone to look after them especially because they're at work and I just need to do something with them erm and I'm going to go with the easier option of just conveying them and let the A&E sort it out. (p. 3) There was a big investment in a new urgent care centre locally but they won't take people who've had a seizure. I've had patients…in a postictal state who need maybe half an hour until they come round…but there's this crazy idea that if somebody's had a seizure then they need to have a CT scan…Sometimes there is no alternative but to take them to ED. (p. 8)

P, participant number.

#### Theme 1: need for relevant historical information to guide care and conveyance decisions

Participants reported that the most common seizure scenario paramedics encounter is a patient who is no longer seizing:

We deal with the aftermath. By the time we get there the patient is either in the postictal stage or recovered. It's very rare that we need to physically treat a seizure. (p. 17)

Participants noted the key clinical question they needed to answer when attending a seizure patient was what the seizure indicated. Was it, for example, a manifestation of an established epilepsy diagnosis, and if so, how ‘normal’ it was? All said that answering this question could be challenging. A number of reasons for this were identified. One was the lack of information paramedics had on patient medical history. Paramedics do not routinely have access to medical records and so often relied on information provided by significant others accompanying the patient. Patients were often in postictal states and required time before they themselves could reliably respond to questions. This led to information gaps when time-critical decision-making was potentially needed.

Identification cards and jewellery were noted to be available for people with epilepsy, but finding a person with one was described as ‘like striking gold’ (p. 1). Contacting the patients’ general practitioner (GP) was another way information might be obtained. However, the utility of this mechanism was described as limited since it depended on knowing who the GP was, how accurate/comprehensive the GP's record was and how quickly the information could be obtained.

#### Theme 2: perverse incentives to convey to ED caused by time pressure/performance requirements

Having to wait to obtain information was important as most participants identified an additional challenge—namely, that paramedics were expected to spend only 15–30 min ‘on scene’ and that non-conveyance to ED was not something the service was currently incentivised to do:

The only measure of success for the ambulance service is how quickly we respond. …We're not at all judged on how we've dealt with the individual. … Every time we miss our target for a priority call, there's a financial implication … so you find yourself under a lot of pressure to get back on the road. (p. 7)

In some localities, participants reported that individuals were assessed according to their own response times. Other participants described how the time pressures were more subtly communicated. Some felt able to withstand the pressure and not let it influence their care decisions. Others could not:

As an individual practitioner, if I don't hit my times I'll be pulled in by my manager so it does pile the pressure on. …Time pressures are maybe what's forcing crews to think, ‘do you know what, just put them on the truck and take them to hospital’. (p. 8)

In explaining what influence time pressures have on paramedics’ decisions, participants explained how because travel times to EDs were often greater in rural areas, conveyance was less appealing as a care management strategy there and likely accounted for some geographic variation between areas.

#### Theme 3: knowledge gaps and uncertainty about postictal care

Beyond the difficulties of obtaining information on a patient's medical history and the time pressures, participants identified an additional issue of importance that they believed was of concern, namely that many paramedics do not feel sufficiently trained to confidently manage seizures. How to care for patients who were no longer seizing was identified by many as being an area where knowledge was particularly low:

We end up getting called but normally the patient has stopped seizing by the time we arrive. …There is though this sort of anxiety if they arrive on scene and the seizure has stopped. It's a big grey area…where the patient presentation is slightly beyond what you're comfortable with you take the patient to ED because it's a sort of default you know. We hand the patient to a doctor to be checked over as a safety net. (p. 1)

The limited attention given to seizures within basic paramedic training and a lack of subsequent training opportunities was said to explain this:

Training on managing seizures becomes part of the neurological syllabus so you might get a couple of hours, if that. … The focus is really on the emergency side of things. (p. 6)

Most reported how lack of training was compounded by limited guidance being available on how to manage patients who were no-longer seizing. JRCALC's guidelines were said to be limited:

The national ambulance guidance is very much based on the acute emergency and not on the urgent phase or the less urgent cases…that's the bit that frustrates me, it's all very much based on conveying to ED. (p. 15)

When ‘on-scene’ participants said they could, in principle, access telephone support or request a further clinician on-scene. Neither resource was identified by participants as being used when managing seizures. The reason for this was said to be because staff knew the resource was limited and so reserved for the most serious of incidents. Several participants felt better access to senior or centralised specialist advice might improve management of seizure patients. Conversely, others highlighted that such resources might not be the most effective approach. One said:

You might say the clinical support desk is under used…I would argue it's more a case of actually, if they were of the right mind set, had the right training, felt supported then they would just be able to make that decision themselves and they wouldn't need to ring.… (p. 3)

#### Theme 4: limitations in care pathways and need for patient-centred care

The final theme was that there can be a lack of care pathways available to paramedics other than conveyance to ED. This could mean that even if a paramedic had managed to secure information on a patients’ medical history and felt confident in interpreting that the seizure a postictal patient had experienced did not require emergency medical attention, conveyance to ED still often remained the only option available to them.

Participants identified that it was a place of safety where the patient could be left to rest and if incontinent, obtain a change of clothes, that was typically needed when managing someone who had experienced a seizure:

A lot of the time they're epileptic, they know what's happened. They know they've had a seizure and they just want to sleep. (p. 5)

Just over half (n=10) of the participants’ employing service had introduced the Paramedic Pathfinder tool that aims to facilitate appropriate non-conveyance. One summarised the tool as follows:

It allows crews to follow in a methodical way the patient presentation and to make a decision based on acuity… As the patient presents less poorly you work your way down until you find a natural point where the patient sits and then it advises you of where to go. (p. 2)

Participant perceptions about the helpfulness of the tool were mixed. A minority said it offered reassurance and structured decision-making. Most participants held an opposing view. They were sceptical about the tool's value and it was seen to threaten practitioner autonomy and skill development:

I mean we have got things in place for staff called Pathfinder where we can direct them down another route rather than just taking the patient to hospital and ie, used, but I think if we gave staff a greater understanding or more knowledge and skills relating to the treatment of people with epilepsy then it might reduce the number of people that have to go with that condition. (p. 10)

Indeed, participants from a number of localities articulated beliefs about how the tool was being misconstrued and leading to more, rather than less, conveyances to ED.

Participants said Paramedic Pathfinder and JRCALC's national guidance alluded to alternative care pathways for adults with established epilepsy who had experienced uncomplicated seizures. This included discharging patients at scene. Discharging the patient was often said to be difficult though, especially when the seizure had occurred in a public place, because the patient needed to be left in the care of a responsible person who could monitor their recovery.

Participants said another reason why a patient might not be discharged related to perceived risk; both to the patient if an adverse event occurred and to the paramedic in terms of their professional status:

There's this kind of fear that some staff may have, well if we leave this patient at home and something happens we're going to get kind of criticised for that because we should of taken the patient to hospital. So they err on the side of caution. There's a saying, ‘you can't get disciplined for taking someone to hospital’. (p. 10)

Alternative options to discharging the patient at the scene involved referring the patient to their GP or an urgent care centre. Both options were described as being rarely available. This frustrated some participants as it meant they could not deliver the care many patients wanted:

The reality is, do most patients that are known epileptics want to go to hospital? Absolutely not… they're sick of the place they don't want to go to hospital … they want to just be managed in an unfussed manner and then allowed to go on their way … but you know a lot of them aren't getting that opportunity … you'll see them waking up in ED and say ‘What on earth have you brought me here for?’ (p. 8)

## Discussion

To help limit the demand on ED within the UK, the ambulance service is expected to avoid ‘unnecessary’ conveyances to EDs.^50^ Most seizure patients attended to by paramedics do not require ED. Our results though suggest paramedics are not currently working within a system that facilitates *not* taking these persons to hospital. Rather, organisational, structural, professional and educational factors create a momentum for them to transport these patients to ED. These shall now be discussed.

### Need for more information of patient's medical history and support

One factor was the lack of ‘on scene’ access paramedics have to information about a patient's medical history. Lack of access to such information is a persistent challenge for paramedics. In the case of seizures, this information though is critical for treatment decisions as it helps the paramedic judge the normality or otherwise of the presentation. Most seizures attended to by paramedics will not indicate a life-threatening condition^1^ and most patients will return to their baseline level of health without medical intervention. Our results show how a lack of access to information about a person's medical history meant paramedics are often unable to differentiate between those who do and do not require emergency medical attention. As such, transporting seizure patients to ED remained the default option for many.

Our results underline the need for new systems to allow more data sharing with the ambulance service. In support of this, Zorab *et al*^51^ presented paramedic participants with a range of hypothetical scenarios. As expected, management decisions regarding convulsions, in particular conveyance, changed by providing additional information. There is research currently underway in the UK which aims to develop a system to increase the information that paramedics have about a seizure patient they attend;^52^ it is however in its infancy. In another project, our group is focusing on people with epilepsy who frequently visit ED.^53^ As part of a seizure management first-aid intervention they and their carers receive, they are being given time and support to develop an emergency care plan to carry with them or have on their ‘smart phones’ to help paramedics. The proportion of people with epilepsy who currently have such plans is low.^54^

### Perceptions about a lack of organisation backing

Further facilitating conveyance to ED were concerns about a lack of organisational support if something adverse happened. This was identified by our previous, regional study.^16^ It has also been acknowledged in studies from the wider literature.^55^ ^56^ Future research should explore how staff can be made to feel more supported. Our participants said services might do this by better disseminating information about cases where staff have been supported when an unexpected event had occurred. What may also be reassuring is if paramedics could be confident, there was a process of review/follow-up for those patients they left.

### Time pressure

Time was described as being often needed when managing a seizure patient. Be it to source information on the patient's history, to transport them home or to observe and decide how well they were recovering. Operationally, time was something paramedics were not afforded and this limited care options.

One performance target which dominates the funding of ambulance services is how quickly they respond to urgent and immediately life-threatening incidents. Services have a target of being on scene within 8 min in 75% of these cases.^57^ With calls to the ambulance service rising,^18^ services are finding it increasingly difficult to achieve this.^58^ Paramedics are aware of this and feel pressure to spend only short periods of time with a patient at the scene.

The limited time participants’ reported paramedics feel able to spend on-scene before transporting someone to ED aligns Dickson *et al*'s^1^ recent findings. They examined records of one regional service's management of seizures. The mean time ‘on scene’ was 27 min. Such a brief window will not afford a paramedic sufficient time to judge if someone with epilepsy has made an uncomplicated recovery or the time for someone to recover to be able to care for themselves and be permitted to be discharged. Even after a complex-partial seizure, it can take 1–2 hours before someone approaches their baseline cognitive level.^59^

The perverse effect that time-based targets might have on the quality of care offered by paramedics has been previously speculated.^60^ ^61^ Our findings provide a detailed example of how this happens in the case of seizures and adds to calls for the development of and greater use of outcome measures for the ambulance that incentivise care that is high quality and cost-effective for the health service as a *whole*.^57^ ^62^ ^63^

### Confidence in seizure management

An added complexity was that paramedics confidence was often low in being able to know when it was and was not safe to leave a seizure patient at the scene. Participants said scant attention was given to seizure management, particularly the postseizure state, within basic paramedic training and postregistration training opportunities.

Traditionally, paramedic training has focused on the assessment and procedures for treating patients with life-threatening conditions. There is a drive to now revise its content, so paramedics are better prepared to perform the evolved duties expected of them. New curriculum guidance has recently been developed for higher education providers.^64^ It does not specify what clinical presentations should be covered, nor to what extent. It does though state paramedics need to be able to “understand the dynamic relationship between human anatomy and physiology. This should include all major body systems with an emphasis on cardiovascular, respiratory, nervous, digestive, endocrine, urinary and musculoskeletal systems” (p. 21). And, that they should be able to “evaluate and respond accordingly to the healthcare needs of patients across the lifespan who present with acute, chronic, minor illness or injury, medical or mental health emergencies” (p. 35). It remains to be seen how this will be translated by institutions and what learning students will receive on seizures.

We would acknowledge here that any curriculum would need to reflect the workload of paramedics and there will be other presentations competing for slots within it. Dickson *et al*'s^1^ evidence could be helpful here in prioritising attention. In examining 1 year of calls to a regional UK ambulance service, they found calls relating to *suspected* seizures were the seventh most common, accounting for 3.3% of calls.

### Guidance documents and tools

It is important to also consider what can be carried out to support already qualified paramedics. Our second paper describes their learning needs and how these might be addressed (FC Sherratt, *et al*. BMJ Open submitted). Another important issue for them though relates to guidance. Participants said the lack of detailed national guidance on the management of postictal patients compounded problems. Only 230 of the 1800 words dedicated to the management of convulsions in adults within JRCALC^19^ relate to the management of such a state. Our findings suggest this section warrants revision. Having said this, evidence from medicine shows changing and revising guidelines does not necessarily mean practice will change,^65^ ^66^ and so the impact of any changes to JRCALC should be evaluated.

Paramedic Pathfinder is a new tool and minimal evidence on its utility is available.^20^ Most of our participants said it was not helpful in promoting care quality for seizure patients. In no way, did it address the difficulties and challenges they reported. Indeed, one criticism was that the alternative care pathways it directed them to did not exist in reality. Last year eight health vanguards were initiated in England. These seek to implement and explore new ways that different parts of the urgent and emergency care sector can work together in a more coordinated way.^67^ These might provide a mechanism by which to bring about the improved access to alternative care pathways that paramedics need.^62^ This awaits to be seen.

### Strengths and limitations

This is the first study to explore from a national perspective paramedics’ views and experiences of managing seizures. The previous study on the topic^16^ and indeed qualitative studies looking at paramedics practice more generally (eg,^68–70^) have recruited from only single sites. Paramedics for this study were recruited from five different services and so it is likely the issues reported do not relate to isolated, local concerns, but reflect practice across the country. The results may also have relevance internationally as countries such as the USA, Australia, Canada and New Zealand have *similarly* organised emergency systems and are also seeking new ways to reduce conveyance rates and ED admissions.^11^ Potential limitations to the study include that it is based on the perceptions and experiences of a self-selecting sample of participants, rather than field observations of what the actual barriers are. The study also did not capture the perspective of associated services providers (eg, urgent care centres, GPs), nor patients and carers. This would have likely provided broader insights on some of the factors which the paramedics identified as being important.^71^ ^72^

## Conclusions

Organisational, structural, professional and educational factors converge to discourage paramedics from considering alternatives to ED transfer when managing people with epilepsy who have experienced an uncomplicated seizure. Efforts are now needed to begin to address these, so as to allow paramedics to deliver care that is in the best interests of patients and the health service as a whole.
